# Exhibitionism, substance use, and genital self-mutilation: A case report of Emotionally Unstable Personality Disorder

**DOI:** 10.1192/j.eurpsy.2026.11072

**Published:** 2026-07-31

**Authors:** A. L. Volpatto, F. Borghi, M. V. Franco Geraldo, C. H. Morais Reis

**Affiliations:** Psychiatry, SMS- Secretaria Municipal de Saúde de São José do Rio Preto, São José do Rio Preto, Brazil

## Abstract

**Introduction:**

Emotionally Unstable Personality Disorder (borderline type) involves emotional instability, impulsivity, and unstable relationships. Impulsivity often leads to risky behaviors such as substance abuse, hypersexuality, self-mutilation, and suicide attempts. When associated with impulse control disorders and substance use, the condition worsens, affecting treatment and functioning. Some severe cases include genital self-mutilation.This report describes a clinical case of Emotionally Unstable Personality Disorder with severe impulsivity, hypersexuality, cocaine abuse, and genital self-mutilation, discussing the diagnostic, therapeutic, and functional challenges

**Objectives:**

To report a complex clinical case of Emotionally Unstable Personality Disorder with severe impulsivity, substance use, hypersexuality, and genital self-mutilation, highlighting the clinical and functional challenges resulting from the association between personality disorders and impulse control disorders.

**Methods:**

This is a clinical case report of a 48-year-old male patient, single, a teacher with a higher education degree. Psychiatric follow-up began in 2019, motivated by abusive cocaine use starting at the age of 37. The clinical history was obtained through interviews, psychiatric records, and patient self-reports. The diagnostic assessment was based on DSM-5 criteria for Borderline Personality Disorder and Impulse Control Disorders.

**Results:**

The patient exhibited severe impulsivity, hypersexuality, substance use, and high-risk behaviors, including exhibitionism and the sharing of intimate images on digital media. Following these episodes, he reported intense regret, followed by severe self-mutilation, such as genital injuries resulting in penile amputation, urethral stenosis, and the need for catheterization. He also reported a history of childhood neglect and violence. He is currently on regular use of Sertraline 100 mg/day, Levomepromazine 15 mg/day, Diazepam 5 mg/day, Naltrexone 50 mg/day, and antiretroviral therapy (HIV-positive). At present, he is abstinent from substances and has had no recent episodes of self-mutilation. Despite partial symptom improvement and increased insight, he continues to experience significant functional impairment, being on medical leave from work, with a vocational rehabilitation plan in progress.

**Conclusions:**

This case illustrates the severity and complexity of comorbid conditions involving personality disorders, impulsivity, substance use, and early trauma. Impulsivity contributes to high-risk behaviors and severe outcomes such as genital self-mutilation. Management requires a multidisciplinary approach involving pharmacological treatment, intensive psychotherapy, and psychosocial support, aiming at harm reduction, functional rehabilitation, and relapse prevention.

**Disclosure of Interest:**

None Declared

